# Serous Tubal Intraepithelial Carcinoma After Neoadjuvant Chemotherapy: A Report of 2 Cases

**DOI:** 10.1097/PGP.0000000000001045

**Published:** 2024-08-22

**Authors:** Iris A.S. Stroot, Leonie Smit, Geertruida H. de Bock, Marise M. Wagner, Mathilde Jalving, Léon C.L.T. van Kempen, Joost Bart, Marian J.E. Mourits

**Affiliations:** * Department of Gynecologic Oncology; † Department of Epidemiology; ‡ Department of Medical Oncology; § Department of Pathology and Medical Biology, University Medical Centre Groningen, University of Groningen, Groningen, the Netherlands; ∥ Department of Pathology, Antwerp University Hospital, University of Antwerp, Edegem, Belgium

**Keywords:** Serous tubal intraepithelial carcinoma, Neoadjuvant chemotherapy, High-grade serous carcinoma

## Abstract

Serous tubal intraepithelial carcinoma (STIC) is regarded as the origin of most high-grade serous carcinomas (HGSC). After a diagnosis of isolated STIC, risk of developing HGSC is substantial. Since surveillance cannot detect HGSC in time to cure the disease, there is no consensus on the optimal treatment after a diagnosis of isolated STIC, but chemotherapy is considered one of the possible strategies. In this case report, we describe 2 women with advanced-stage HGSC treated with 3 cycles of neoadjuvant chemotherapy followed by interval debulking surgery. In both women, histopathological examination showed a complete histopathological tumor response, but a vital STIC was found in both cases. The 2 cases presented here indicate that STICs may not respond to chemotherapy. Further research focused on the underlying biology and chemosensitivity of STIC, as well as the effectiveness of treatment to prevent HGSC in case of isolated STIC, is needed.

Serous tubal intraepithelial carcinoma (STIC) is regarded as the precursor of most (if not all) high-grade serous carcinomas (HGSC). In a study by Steenbeek et al, the 5- and 10-year risk of HGSC after isolated STIC found at adnexal resection was estimated at 10.5% (95% CI: 6.2-17.2) and 27.5% (95% CI: 16.6-43.9), respectively^1^. Therefore, a diagnosis of isolated STIC is currently recognized as one of the most important risk factors for the subsequent development of HGSC^2^. However, there are no guidelines on how women with isolated STIC should be treated to prevent subsequent HGSC. To contribute to the discussion on this topic, we report on 2 patients with a complete histopathological response of advanced-stage HGSC after neoadjuvant chemotherapy (NACT), but in whom STIC was detected in their fallopian tubes removed during debulking surgery. We discuss the possible implications of these findings to add to the current debate on treatment options for women diagnosed with isolated STIC.

## CASE REPORT

**FIGURE 1:**
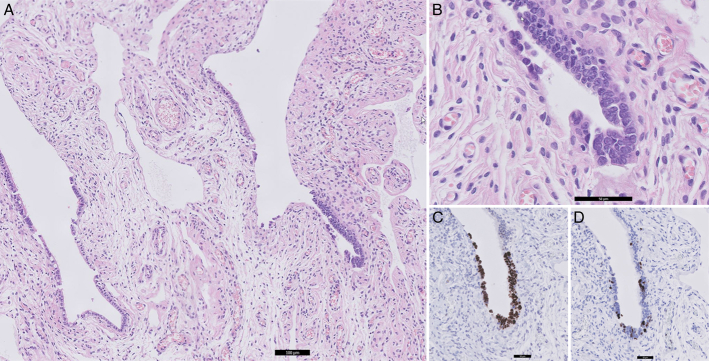
Serous tubal intraepithelial carcinoma located in the right fimbriated end of patient 1 showing a focus of atypical epithelial cells with enlarged nuclei, nuclear pleomorphism, pseudo stratification, and prominent nucleoli. (A). Serous tubal intraepithelial carcinoma of patient 1 at higher magnification (B). Immunohistochemistry with a p53 overexpression pattern of serous tubal intraepithelial carcinoma of patient 1 (C). An increased Ki-67 index of serous tubal intraepithelial carcinoma of patient 1 (26%) (D).

The first case is a 51-year-old woman who presented with dyspnea and pleural effusion. Cytology showed highly proliferative malignant cells with features of adenocarcinoma, likely from HGSC of tubo-ovarian origin, with an overexpression pattern of p53, a Ki-67 index of 70%, and WT-1 and PAX-8 positive cells. After 2 cycles of NACT with carboplatin and paclitaxel, the CT scan showed a significant decrease in ascites, pleural effusion, omental cake, and peritoneal depositions. After the third cycle of chemotherapy, interval debulking surgery was performed, removing uterus, adnexa, omentum, and small peritoneal lesions, resulting in a complete debulking. Both fimbriae were entirely embedded for histopathological examination, together with fallopian tubes and ovaries. Representative slides of the uterus and omentum were made. Histopathological examination showed no vital tumor in the removed tissues. However, both fimbriated ends of the fallopian tubes revealed a focus on atypical epithelial cells with enlarged nuclei, nuclear pleomorphism, pseudo stratification and prominent nucleoli. The foci both showed overexpression of p53 and increased Ki-67 index (26%), consistent with STIC (Fig. 1). No evident effect of chemotherapy was observed in the STIC lesions.

**FIGURE 2:**
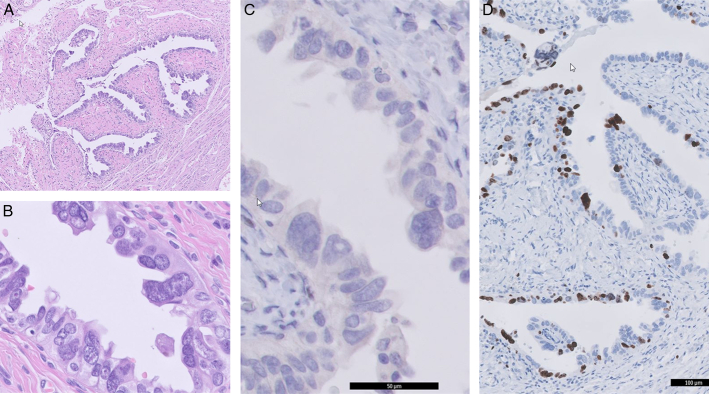
Serous tubal intraepithelial carcinoma located in the left fimbriated end of patient 2 showing atypical epithelial cells with enlarged, irregular nuclei (A). Serous tubal intraepithelial carcinoma of patient 2 at higher magnification B). Immunohistochemistry with a p53 null-mutation pattern of serous tubal intraepithelial carcinoma of patient 2 (C). An increased Ki-67 index of serous tubal intraepithelial carcinoma of patient 2 (34%) (D).

The second case is a 79-year-old woman who presented with abdominal distention and fatigue. A CT scan showed ascites, peritoneal depositions, and omental cake. A omentum biopsy showed cells with a null-mutation pattern of p53, positive WT-1, positive PAX-8, and a Ki-67 index of 100%, suggesting HGSC of tubo-ovarian origin. After 2 cycles of NACT with carboplatin and paclitaxel, a good clinical, biochemical, and radiologic response was observed. After 3 cycles, interval debulking surgery was performed, removing omentum, uterus, adnexa, part of the rectosigmoid, and small peritoneal lesions, resulting in complete debulking. Both fimbriae were entirely embedded for histopathological examination, together with fallopian tubes and ovaries. Representative slides of the uterus, part of the rectosigmoid, and omentum were made. Histopathological assessment of the removed tissue revealed no vital tumor residue. However, the fimbriated end of the left fallopian tube showed 2 foci of atypical epithelial cells with enlarged, irregular nuclei with a null-mutation pattern of p53 and an increased Ki-67 index (34%), characterized as STIC (Fig. 2). No evident effect of treatment was observed in the STIC lesion.

## DISCUSSION AND CONCLUSION

In this case report, we report on 2 cases with a complete tumor response in whom a STIC was found following NACT. STIC has been established as the most important risk factor for subsequent HGSC development^1^. A carcinogenic sequence has been proposed, with a *TP53* mutation as initiation and, through the accumulation of cancer-promoting events, progression to STIC and eventually invasive HGSC^2^. Although the persistence of STIC in the fallopian tube following NACT has been described before by Colón et al^3^, this is the first report of 2 patients with advanced-stage HGSC in whom vital STIC lesions were noted after treatment with NACT, while HGSC showed a complete histopathological response.

The women described here both received combination chemotherapy with carboplatin and paclitaxel, which primarily affects rapidly proliferating cells. The Ki-67 index of both STICs was significantly lower than that of the tumors sampled before the NACT. This difference in the Ki-67 index may explain the observed lack of treatment effect in the STICs, whereas the tumor showed a complete response. Colón et al made a similar observation, as they measured a Ki-67 index of 30% and 35% in the 2 of the STICs they reported following NACT^3^.

Although it is possible that the Ki-67 index of the STICs changed in response to NACT, as has been described for ductal carcinoma in-situ of the breast^4^, it is important to note that the Ki-67 index was clearly higher than that of the surrounding histologically normal mucosal fallopian tube epithelium, indicating that these STICs are not dormant. It has been recognized that the proliferation rate in STICs can be highly variable (10%–90%)^2^. Therefore, it is possible that a subset of STICs can be sensitive to chemotherapy. To further investigate the significance of our findings, more research on the phenomenon of STIC and the different proliferation indices is needed to understand the chemosensitivity of STICs.

There are several small observational studies in which women with isolated STIC were given chemotherapy as a preventive measure against developing HGSC^5–7^. At the end of the follow-up, none of the women in these studies developed HGSC. However, this does not prove the added value of chemotherapy since the numbers were small, and the majority of surgically removed STICs are not followed by HGSC later in life^1^. Thus, many uncertainties remain about the underlying biological mechanism, the course of progression from STIC to HGSC, and the chemosensitivity of STICs.

Although we describe only 2 cases of persistent STIC after complete remission of HGSC after NACT, these 2 well-documented cases provide valuable information and further direction for studying treatment options for STIC, as currently, there are limited opportunities to study the biological course of STIC due to the rarity of the disease. Given the fact that there is a substantial risk of HGSC after a diagnosis of isolated STIC and that there are no methodologies for early detection or curative therapies, further research focused on the biology of the development of STIC to HGSC, as well as knowledge regarding the efficacy and proportionality of treatment to prevent HGSC after STIC is direly needed.

The observation of the 2 cases with isolated STIC after complete tumor response after neoadjuvant chemotherapy in advanced HGSC may also be significant for the larger group of patients with HGSC and concurrent STIC. It may be hypothesized that a vital STIC detected after NACT is a potential adverse prognostic marker for patients with otherwise complete tumor response, which deserves further investigation. In addition to the systematic examination of the fallopian tubes of all patients with HGSC by preferably applying the SEE-FIM protocol (Sectioning and Extensively Examining the FIMbriated end)^8^, it is essential that pathologists systematically document the presence of STIC after NACT found in the pathology report, either in the presence of residual viable tumor or in the case of complete pathologic response. Ultimately, this systematic documentation of residual vital STIC, which many pathologists may have observed without considering the potential scientific implications, may contribute to further knowledge about the prognostic impact of STIC found after NACT in patients with HGSC.

In conclusion, a STIC can be detected after NACT, even with a complete tumor response. We hope that the description of these 2 cases will contribute to the discussion of desirable management strategies after a diagnosis of isolated STIC and help future studies with systematic documentation of residual STIC after NACT.
